# Improving R Peak Detection in ECG Signal Using Dynamic Mode Selected Energy and Adaptive Window Sizing Algorithm with Decision Tree Algorithm

**DOI:** 10.3390/s21196682

**Published:** 2021-10-08

**Authors:** Zubaer Md. Abdullah Al, Keshav Thapa, Sung-Hyun Yang

**Affiliations:** Department of Electronic Engineering, Kwangwoon University, Seoul 01897, Korea; kshavthapa@kw.ac.kr (K.T.); shyang@kw.ac.kr (S.-H.Y.)

**Keywords:** QRS detection, ECG interpretation, biomedical signal processing, machine learning, intelligence medical device

## Abstract

R peak detection is crucial in electrocardiogram (ECG) signal analysis to detect and diagnose cardiovascular diseases (CVDs). Herein, the dynamic mode selected energy (DMSE) and adaptive window sizing (AWS) algorithm are proposed for detecting R peaks with better efficiency. The DMSE algorithm adaptively separates the QRS components and all non-objective components from the ECG signal. Based on local peaks in QRS components, the AWS algorithm adaptively determines the Region of Interest (ROI). The Feature Extraction process computes the statistical properties of energy, frequency, and noise from each ROI. The Sequential Forward Selection (SFS) procedure is used to find the best subsets of features. Based on these characteristics, an ensemble of decision tree algorithms detects the R peaks. Finally, the R peak position on the initial ECG signal is adjusted using the R location correction (RLC) algorithm. The proposed method has an experimental accuracy of 99.94%, a sensitivity of 99.98%, positive predictability of 99.96%, and a detection error rate of 0.06%. Given the high efficiency in detection and fast processing speed, the proposed approach is ideal for intelligent medical and wearable devices in the diagnosis of CVDs.

## 1. Introduction

**Figure 1:**
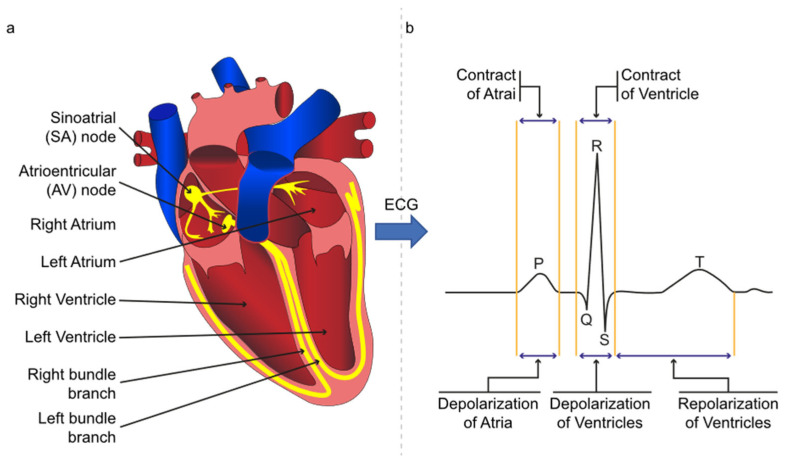
(**a**) The yellow line represents the electrical flow in the heart; (**b**) ECG signal with P, QRS, and T waves.

Cardiovascular diseases (CVDs) cause 31% of global mortality [1]. The burden of CVDs and shortage of physicians will increase to 57% and 42% worldwide by 2034 [2]. In addition, the median accuracy of ECG interpretation is only 54% for all training levels of practicing physicians and cardiologists [3]. The detection of R peaks is the fundamental step for ECG analysis. ECG signal is a non-linear and non-stationary physiological signal that represents the heart’s electrical activities. It is measured by electrodes set on the patient’s skin, forming the Einthoven triangle. Six fiducial points in the ECG signal, i.e., the P, QRS, T, and U, represent heart events during the relaxation or contraction [4] and are presented in Figure 1. The QRS wave is the vital feature for ECG analysis, and the R is the tallest peak in the QRS waves [5]. The ECG morphology varies from person to person, even at a different time for the same individual. In addition, the ECG is generally suffering from power line noise, muscular noise, electrode contact noise, and baseline wander [6]. These factors have added difficulties in R peak detection.

Several methods have been reported for detecting R peak in ECG signal in recent decades, based on Fourier Transform (FT) [7], Short-Time Fourier Transform (STFT) [8,9,10], Wavelet Transform (WT) [11,12,13,14,15,16,17], Empirical Mode decomposition (EMD) [18], adaptive thresholding [5,14,19,20,21], machine learning [22,23], and neural networks [4,24,25,26]. However, the detection efficiency varies with processed signal quality and extracted features [3,5,7,8,9,10,11,12,13]. Adaptive thresholding methods suffer from the miscalculated threshold due to the noise events and require a certain number of previous data points to predict the threshold [5,19,27]. Fourier Transform-based methods collapsed to acquire an accurate frequency spectrum due to the ECG signal’s non-linear and non-stationary characteristics [27]. Short-Time Fourier Transform computes a better frequency spectrum within a window, but a constant window size dimmed to adopt the signal’s characteristics over time [8]. Wavelet Transformation computed the spectrum based on a wavelet, and the wavelet frequencies strictly define the spectrum [11,13,14,28]. Empirical Mode Decomposition and Hilbert Transform quantify the spectral density adaptively, but the recursive decomposition approach increases the computational time complexity of the system [18].

This study proposed DMSE, AWS, and RLC algorithms to improve R peak detection efficiency in real-time. The DMSE algorithm is derived from variational mode decomposition (VMD) [29] and Hilbert Transform (HT) [30], and improved in energy and frequency estimation. The AWS algorithm is derived from the peak detection algorithm and enhances the performance of Region of Interest (ROI) estimation. The R location correction (RLC) algorithm is a search algorithm and progresses in the accuracy of the R location. As a result of these improvements, this study achieved competitive accuracy, sensitivity, and specificity in automatic R peak detection.

## 2. Materials and Methods

### 2.1. ECG Signal Dataset Overview

This study used the MIT-BIH arrhythmia database from the physionet site [31]. It was the first generally available set of standard test materials for evaluating arrhythmia detectors, and the arrhythmia database has 48 two-channel ambulatory ECG recordings of 30 min each. These signals are sampled at 360 Hz and have a resolution of 11 bits over a spectrum of ten millivolts. This study used the first lead of all 48 records for training, validation, and testing the proposed method’s performance in the analysis phase. There are a total of 112,647 annotated labels. Annotations are two types, i.e., beat annotation and non-beat annotation. This study used 18 types of MIT-BIH beat annotations and considered each beat to have an R peak. There is a total of 109,494 QRS beats in the MIT-BIH arrhythmia database. 

### 2.2. Methods 

**Figure 2:**
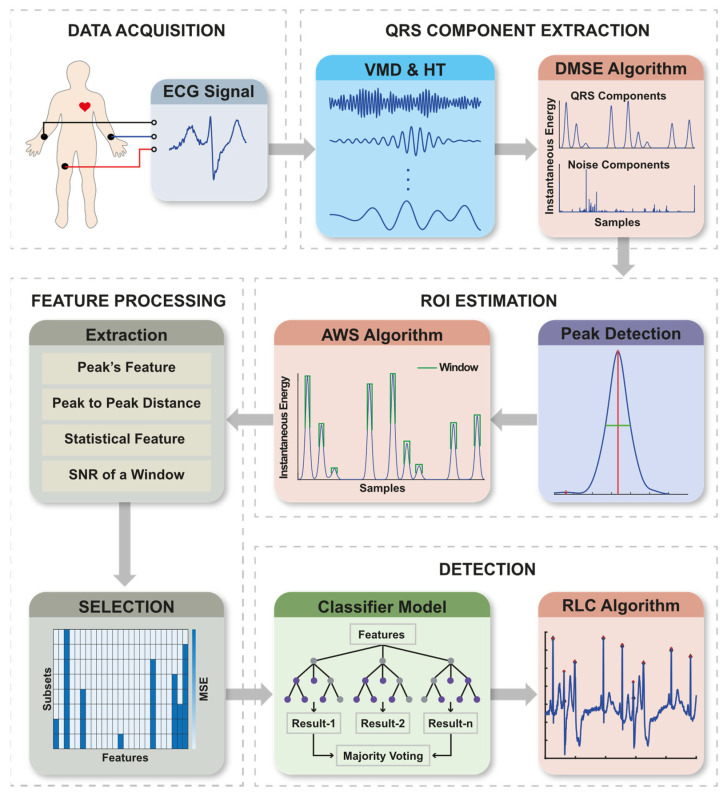
Method’s workflow of R peak detection and its sub-systems.

This proposed method consists of five significant stages: (a) Data Acquisition, (b) QRS Component Extraction, (c) Region of Interest (ROI) estimation, (d) Feature Processing, and (e) Detection. Figure 2 illustrates the block diagram of the proposed method and its sub-systems. In addition, Graphical User Interface (GUI) software is developed to operate the proposed method in real-time.

#### 2.2.1. Data Acquisition

This study interfaces the AD8232 EKG sensor (SparkFun Electronics, Niwot, CO, USA) module with an Arduino board (Arduino, Scarmagno, Italy). A Jetson Nano micro-computer (Nvidia Corporate, Santa Clara, CA, USA) receives data from Arduino at a configurable sampling rate via serial port.

#### 2.2.2. QRS Component Extraction

The QRS Component Extraction section decomposed the ECG signal into modes and computed the Hilbert spectrum using variational mode decomposition (VMD) and Hilbert Transform (HT). Then the dynamic mode selection algorithm determines the QRS components adaptively.

##### VMD and HT

The VMD decomposed a signal $f\left(t\right)$ into K modes $u_{k}\;\left(k=1,2,3,\ldots ,K\right)$. These modes are called VMFs, and a VMF is defined as amplitude-modulated frequency-modulated (AM-FM) signals, written as Equation (1),

(1) $$u_{k}\left(t\right)\;=A_{k}\left(t\right)cos\left(\varphi_{k}\left(t\right)\right)$$

where $u_{k}\left(t\right)$ is a VMF, $A_{k}\left(t\right)\;$ is a nonnegative envelope, and $\varphi_{k}\left(t\right)$ is non-decreasing phase function.

The complex conjugate $y\left(t\right)$ of any real-valued function $x\left(t\right)$ of $L^{p}$ class (Lebesgue spaces) can be calculated by Titchmarsh theorem defined in Equation (2),

(2) $$ℋ\left(x\left(t\right)\right)\;=\frac{1}{\pi }\;PV\int_{-\propto }^{\propto }\frac{x\left(\tau \right)}{t-\tau }d\tau$$

in which the PV indicates the principal value of the singular integral.

With the Hilbert Transform, the analytic signal is defined in Equation (3),

(3) $$z\left(t\right)\;=x\left(t\right)\;+\;iy\left(t\right)\;=a\left(t\right)e^{-j\theta \left(t\right)}$$

where $a\left(t\right)\;=\sqrt{x^{2}+y^{2}}$ and $\theta \left(t\right)\;=\arctan\left(\frac{y}{x}\right).$

Here, $a\left(t\right)$ is the instantaneous amplitude and θ is phase function. The instantaneous frequency and energy are given by Equations (4) and (5), respectively,

(4) $$\omega_{inst}=\frac{d\theta }{dt}$$

(5) $$E_{inst}={\left|a\left(t\right)\right|}^{2}$$

##### Dynamic Mode Selected Energy (DMSE)

QRS waves were observed within a frequency band, i.e., $\omega_{B}\to \left(f_{l},\;f_{h}\right)$. The mean and standard deviation of each VMF’s instantaneous frequency is used to select the modes dynamically. The upper and lower frequency scale of each VMF were defined by the spread from mean frequency and limited by the standard deviation. If both degrees’ intersection is within $\omega_{B}$, those VMFs were considered as QRS components. VMFs’ selection process is presented with Equation (6),

(6) $$k_{s}=\left\{\begin{array}{c} true\\ false \end{array}\begin{array}{c} \;\;\;\;\{(\mu^{\omega_{inst_{k}}}-\sigma^{\omega_{inst_{k}}})\geq f_{l}\}\wedge \{(\mu^{\omega_{inst_{k}}}+\sigma^{\omega_{inst_{k}}})\leq f_{h}\}\;\;\\ otherwise \end{array}\right.$$

It was also observed that some data points in the chosen VMFs included frequencies outside the $\omega_{B}$, causing an unexpected rise or fall at the edges. If instantaneous frequency of a data point is out of $\omega_{B}$, then those data points are detected as noisy. It is presented in Equation (7),

(7) $$\omega_{r}=\left\{\begin{array}{c} 1\\ 0 \end{array}\;\;\;\;\begin{array}{c} \left(f_{l}\leq \;\omega_{inst}\right)\;\wedge \;(\omega_{inst}\leq f_{h})\\ otherwise \end{array}\right.$$

The elementwise multiplication of $\omega_{r}$ with instantaneous energy and frequency of a selected VMF only set the unnecessary data to 0. Then, the integral of all necessary data points of instantaneous energy and frequency is computed by Equations (8) and (9). $R^{e}$ and $R^{\omega }$ accommodate energy and frequency of QRS components.

(8) $$R^{e}=\overset{k_{s}}{\underset{k_{s}=1}{\sum }}\omega_{r}{}_{k_{s}}⊙E_{inst}{}_{k_{s}}$$

where $k_{s}$ is the number of chosen VMF.

(9) $$R^{\omega }=\overset{k_{s}}{\underset{k_{s}=1}{\sum }}\omega_{r}{}_{k_{s}}⊙\omega_{inst}{}_{k_{s}}$$

where $k_{s}$ is the number of chosen VMF.

To obtain the noise component, this study inverts the $\omega_{r}$ and multiplies with instantaneous energy, and finally, the integral of all noise data points is computed with Equation (10).

(10) $$R^{n}=\overset{k}{\underset{k=1}{\sum }}\neg \omega_{r}{}_{k}⊙E_{inst}{}_{k}$$

where k is the number of VMF.

**Figure 3:**
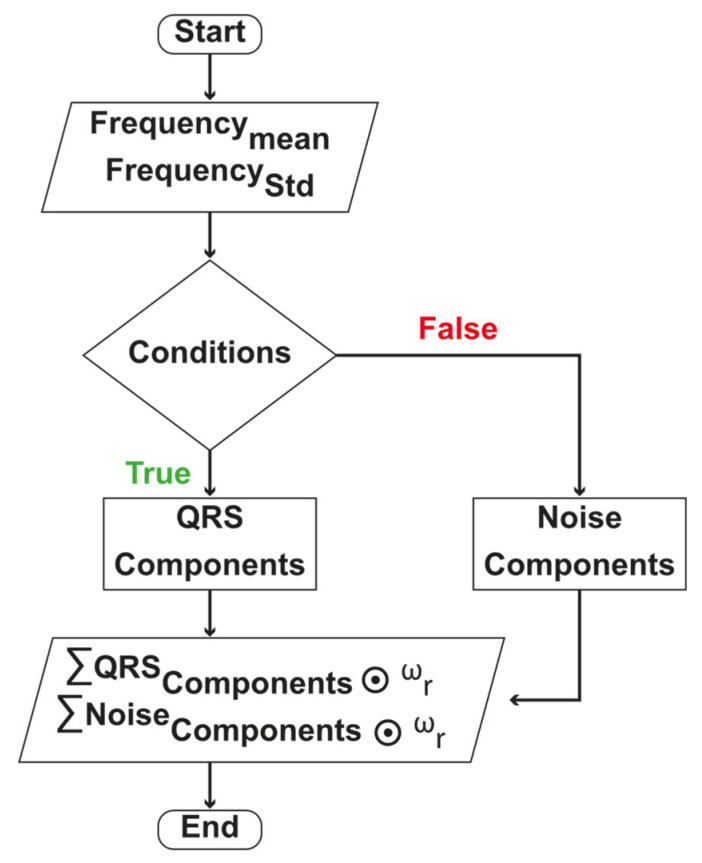
DMSE algorithm flow chart.

The flow-chart of DMSE algorithm is presented in Figure 3.

#### 2.2.3. ROI Estimation

The ROI was estimated from the integral function of energy in QRS components. The AWS algorithm was developed based on local peaks to find ROI adaptively. A peak finding algorithm detects the local peaks and its height, prominence, and width. To compute the ROI, we kept the peak’s location at the center and spread it to the left and right sides up to the peak width. The ROI window is adaptively computed with Equations (11)–(14).

(11) $$QRS_{W_{i}}=\frac{Peak_{width_{i}}}{2}$$

where $Peak_{width_{i}}$ is the width of i th peak.

(12) $$Window_{Start}=Peak_{L_{i}}-QRS_{W_{i}}$$

where $Peak_{L_{i}}$ is the location of i th peak.

(13) $$Window_{End}=Peak_{L_{i}}+QRS_{W_{i}}$$

(14) $$QRS_{Window}=Component\left(Window_{Start}\;:\;Window_{End}\right)$$

**Figure 4:**
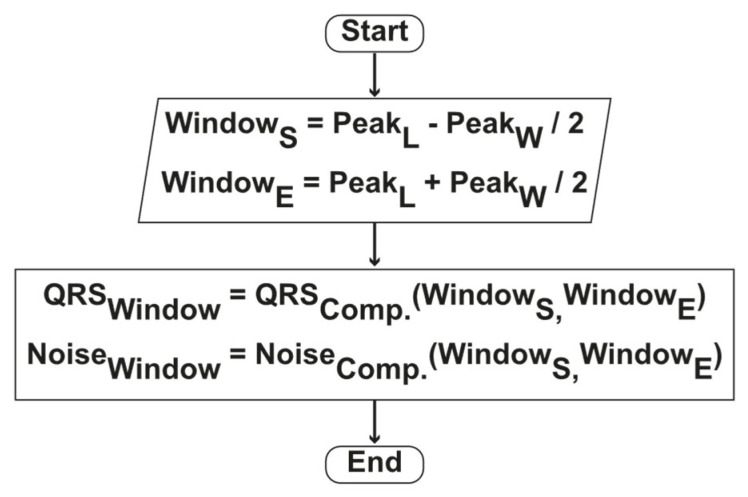
Method’s AWS algorithm flow chart.

The flow-chart of AWS algorithm is presented in Figure 4.

#### 2.2.4. Feature Processing

Classification features were processed in two steps, i.e., (a) Feature Extraction and (b) Feature Selection.

This study calculated twenty-five features, i.e., four features related to peak, two from a distance, eighteen statistical features from each peak’s window, and one from estimated noise. All extracted features are presented in Table 1.

Sequential Forward Selection (SFS) method was used to find relevant features for better classification. SFS is a wrapper-approach method based on the greed search algorithm, reduces N-dimensional feature space to an n-dimensional subspace, where n<N. SFS automatically selects a subset of the most relevant features and removes irrelevant features based on criterion values. Criterion refers to the mean squared error (MSE).

#### 2.2.5. Detection Process

The detection process has an ensemble decision tree (DT) model and RLC algorithm. The model classifies R and False-R. The RLC algorithm finds the exact location of R peaks in the time domain. This study creates a dataset based on detected ROI from the ECG signal database to train, validate, and test the classifier model.

##### Datasets for Classification

This study found that most of the ROIs contain the QRS complex. The QRS containing ROIs are labeled as R otherwise, False-R. The training dataset has been created with 80% of each type of arrhythmia sample from the estimated ROIs. The samples are chosen randomly. The remaining 20% of the data in the ROIs’ dataset is the test dataset. The training dataset is further divided into 10-fold for cross-validation during the training process.

##### Ensemble Decision Tree

A Decision Tree (DT) is a non-parametric and supervised learning algorithm widely used for classification. It classifies by learning decision rules’ reasoning from the features. A tree starts from a root node, splits into branches or sub-trees until it reaches a terminal or leaf node. The root or parent of a leaf node is decided based on the feature approximation constant. This node is called the decision node, and the approximation constant is the rule. The decision tree uses the Gini index to compute the approximation constant. The Gini index computes the relative degree of disparity within the conveyance of classes and ranges from zero to one. Gini Index is computed with Equation (15).

(15) $$I_{G}=1-\overset{k}{\underset{j=1}{\sum }}P_{j}^{2}$$

where $P_{j}$ is the probability of  j class.

Utilizing the Gini index, the most excellent features which contain the foremost information concerning the target are recognized. The dataset and the values of these features are then used to ensure that the target function values at the leaf nodes are as perfect as possible. As a result, a series of decision rules are organized in a tree structure that can distinguish. Therefore, a set of decision rules are arranged in a tree structure capable of classifying. The deeper the tree is, the model is fitter but suffers from an overfitting problem. The ensemble decision tree algorithm has overcome this generalization problem. 

This study trained a total of $2^{n}-1$ decision trees, where n is the number of selected features. Each DT is trained with a different feature set, and 75% of the samples are randomly chosen for training from the training dataset obtained from the ROIs’ dataset. Sixteen DTs have been selected based on the highest validation accuracy. Next, those sixteen DTs are ensembled, and finally, the majority votes decide which ROI contains an R peak.

##### R Location Correction (RLC)

This study observed that some of the detected R peaks are slightly dislocated from the annotated position. This dislocation of peak position creates incorrect results in calculating heart rate. To overcome the dislocation problem, we find the maximum value of a correction window. The position of the maximum value in the signal is considered as an R peak location. It has been observed that the dislocation problem occurs inside a window size of eight. We keep the detected location at the center of the correction window and find the correct location and amplitude as presented in Equation (16).

(16) $$R^{c}=\underset{k}{\max}\left\{R_{k-4}^{e},\;R_{k-3}^{e},\ldots ,\;R_{k}^{e},\;\ldots ,\;R_{k+3}^{e},R_{k+4}^{e}\right\}$$

#### 2.2.6. GUI Software

**Figure 5:**
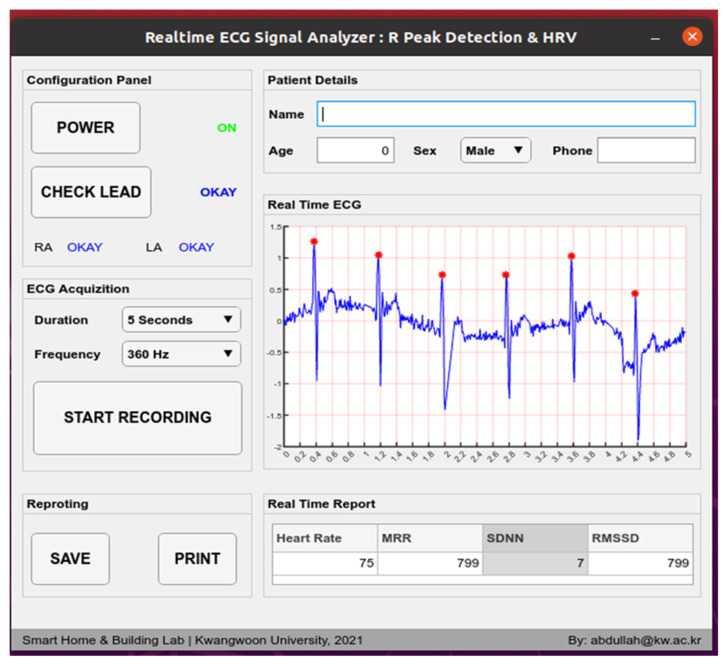
GUI software. The presented ECG in GUI is taken from patient in real-time.

A utility software has been developed with a graphical user interface presented in Figure 5. It can control the power on and off. Lead placement is checkable. Sampling frequency can be configured to 100 Hz, 150 Hz, 200 Hz, and 360 Hz. Time of acquisition is configurable to 5 s, 10 s, 1 min, 10 min, and in monitoring mode. Monitoring mode will be helpful in longtime observation. Realtime ECG with R location marked by an asterisk can be seen. Heart rate, MRR, SDNN, and RMSSD are also presented in the real-time report section. The patient data and model-generated medical information can be saved as a PDF file or printed with this software.

### 2.3. Evaluation Methods

True-Positive (TP), False-Positive (FP), True-Negative (TN), and False-Negative (FN) are the four quantities required to calculate the classification evaluating parameters such as sensitivity, precision, detection error, rate, accuracy, specificity, and recall. TP and TN represent the total number of correctly classified R and False-R. FP and FN indicate the inaccurately detected R and False-R. The evaluation parameters are computed with Equations (17)–(22).

(17) $$Sensitivity=\frac{TP}{TP+FN}$$

(18) $$Precision=\frac{TP}{TP+FP}$$

(19) $$DER=\frac{FP+FN}{TP}$$

(20) $$Accuracy=\frac{TP}{TP+FP+FN}$$

(21) $$Specificity=\frac{TN}{TN+FP}$$

(22) $$Recall=\frac{TP}{TP+TN}$$

The Heart Rate Variability (HRV) is the evaluating parameter for R peak location accuracy. Three types of HRV, i.e., mean of R-R intervals (MRR), the standard deviation of normal-to-normal R-R intervals (SDNN), and root mean square of successive heartbeat interval differences (RMSSDs) are computed with the Equations (23)–(25).

(23) $$MRR=\frac{1}{N-1}\sum_{n=2}^{N}R_{n}^{i}$$

(24) $$SDNN=\sqrt{\frac{1}{N-1}\sum_{n=2}^{N}{\left[R_{n}^{i}-MRR\right]}^{2}}$$

(25) $$RMSSD=\sqrt{\frac{1}{N-2}\sum_{n=3}^{N}{\left[R_{n}^{i}-R_{n-1}^{i}\right]}^{2}}$$

where $R_{n}^{i}$ is the R-R interval between $n^{th}$ and $n^{th}-1$ peak.

### 2.4. Experimental Tools and Environment

This study used a computer with Intel Core i9-9900K CPU (Intel Corporation, Santa Clara, CA, USA), 16 GB of memory, and Ubuntu 20.04 LTS as OS. MATLAB R2020b, Simulink, signal processing and machine learning toolbox are used for signal analysis, machine learning models, simulations, and system modeling. The algorithm is deployed into a practical instrument with an AD8232 EKG sensor module, Arduino board, and Jetson Nano. The desktop-based graphical user interface (GUI) application is deployed to the Jetson Nano micro-computer. This software and device are used to detect real-time R peaks on persons.

## 3. Results and Discussions

The proposed methods’ performance was computed using the MIT-BIH arrhythmia database. For a reasonable evaluation of performance, this study accesses section-wise performance and presented in (a) QRS Component Extraction, (b) ROI estimation, (c) Feature Processing, and (d) Performance of RLC. Finally, the proposed approach is evaluated in comparison to alternative methods in Section 3.5.

### 3.1. Performance of QRS Component Extraction

**Figure 6:**
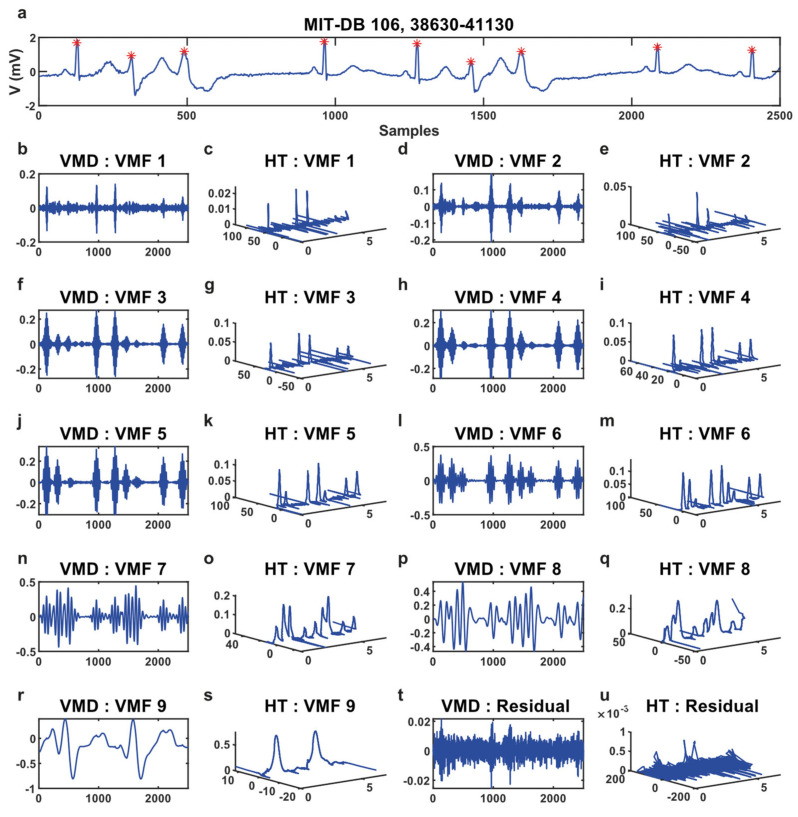
(**a**) Sample ECG signal. * Illustrated the R peaks. (**b**–**u**) Represents the decomposed VMFs and time-frequency-energy of each of the VMFs obtained from Hilbert Transform.

The VMD method decomposes an ECG signal into nine variational mode functions (VMFs), and the Hilbert Transform (HT) converts the modes into time-frequency-energy, shown in Figure 6.

**Figure 7:**
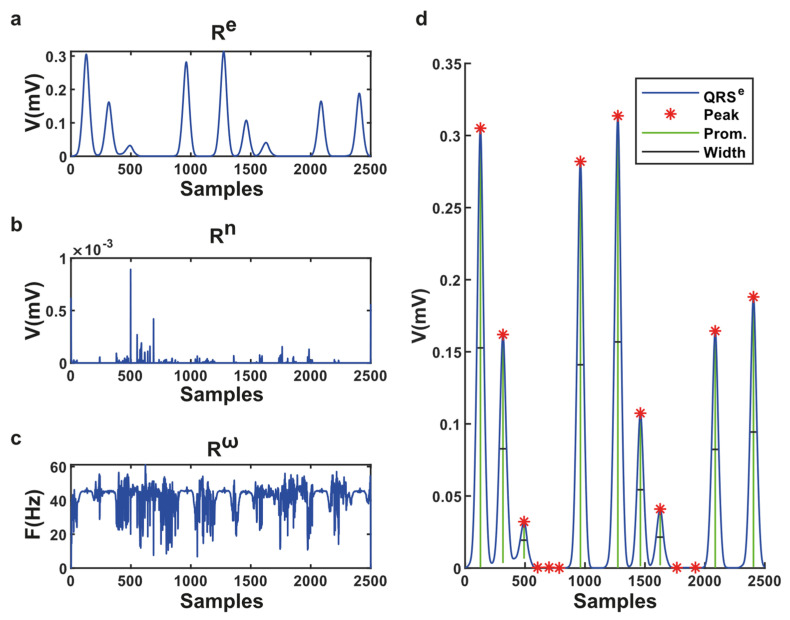
(**a**) QRS component of instantaneous energy; (**b**) The noise of the QRS component; (**c**) The instantaneous frequency of QRS components; (**d**) Detected peaks in the QRS component.

In this example signal (Figure 6a), there are nine QRS complexes. It has been observed that in VMF-6, there are nine prominent peaks with low noises. Therefore, the VMF-6 represents the best time, frequency, and energy for this signal, although it can be different for different ECGs. The DMSE algorithm determines the VMFs which contain the best QRS data; otherwise, noises are considered. It suppresses all unnecessary waves like P, T, and U from the ECG signal presented in Figure 7.

For estimating the DMSE algorithm’s performance, this study counts the real, missing, and false R peaks in the integral function of the QRS component. There are 15 types of heartbeats in the MIT-BIH arrhythmia database and a total of 109,494 beats. Aberrated Atrial Premature, Fusion, Paced, Premature Ventricular Contraction, APC and Normal types suffer a loss of 0.66, 0.12, 0.07, 0.084, 0.039, 0.059%. The rest are nine types of beats and suffer zero loss. Considering the True beat counts, the mean error of the DMSE algorithm is 0.0538%. A total of 22,145 false R peaks were detected. The DMSE algorithm removes 89.887% of P, T, and noise-associated peaks. We present the performance of the DMSE algorithm in Table 2.

### 3.2. Performance of ROI Estimation

**Figure 8:**
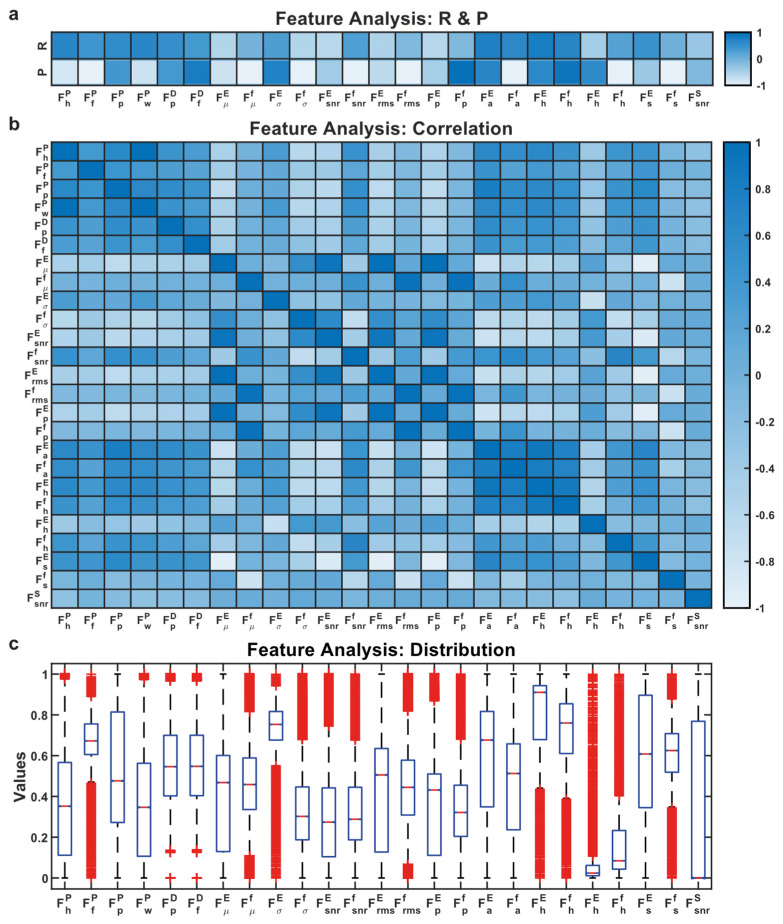
(**a**) R and P represent the Pearson correlation and significance of the features; (**b**) Cross-correlation between features indicates redundancy; (**c**) Distribution of features.

The purpose of the AWS algorithm is to obtain the accurate feature of QRS. Therefore, a feature set with a lower error represents the performance of the AWS algorithm. This approach used the sequential forward selection (SFS) approach for choosing the best features. The SFS method measures the feature importance or criterion one by one and includes it into the selected subsets. An ensemble decision tree algorithm was chosen for classification based on the feature distribution as shown in Figure 8c.

**Figure 9:**
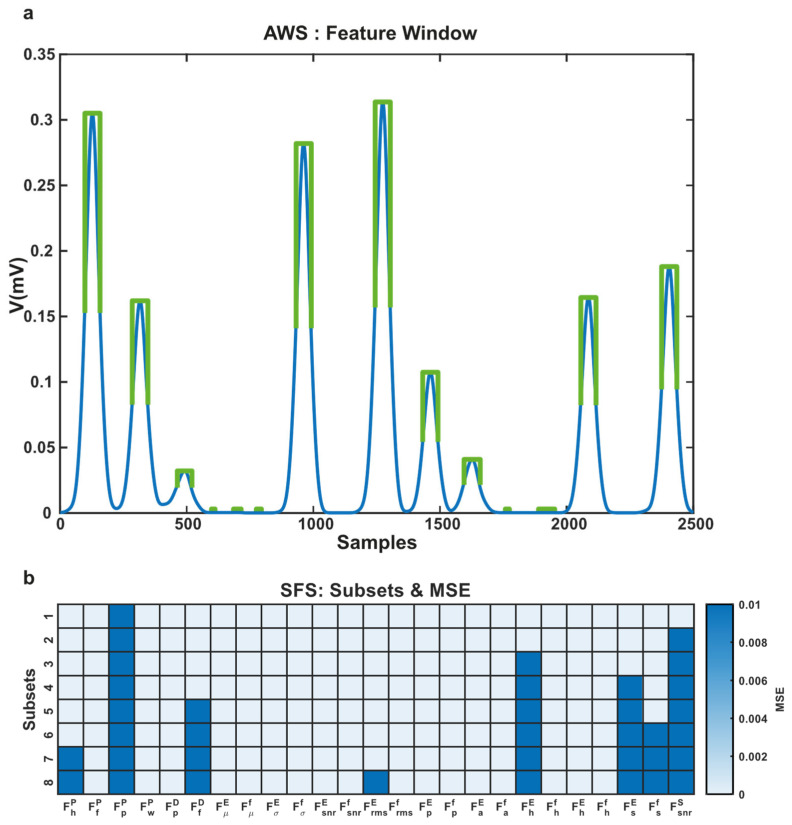
(**a**) The adaptive feature window is presented with green color; (**b**) MSE errors of chosen subsets.

This study has eight subsets of features from twenty-five features. The mean squared error of each subset reveals the performance of each subset. We achieved the lowest MSE 0.001 for the eighth subset and the highest 0.0077 MSE for the first subset. In Figure 9, the adaptive feature window and performance of the AWS algorithm are presented.

### 3.3. Performance of Detection

The sensitivity, specificity, precision, recall, detection error rate (DER), and accuracy are measured for evaluating the classification performance. The sensitivity and specificity represent the ability to classify R and False-R accurately. Precision reports the rate of accurately classified R among all detected R. On the other hand, recall tells the rate of accurately detected R and False-R. DER notifies the ratio of incorrect and correct classification of R and False-R. Finally, accuracy represents the percentage of correct classification. The overall classification performance of the proposed method is measured by the area under the ROC curve (AUC). 

The classifier model is evaluated with a 10-fold cross-validation dataset and test dataset. To analyze the performance in a real-time scenario, 1.5 s long window with 0.5 s overlapping was considered as a sample signal. Thus, R peaks in each signal file of MIT-BIH arrhythmia database are predicted, representing the actual performance of our proposed model.

The confusion matrix and performance evaluating parameters for the 10-fold validation, test, and MIT-BIH are presented in Table 3 and Table 4.

### 3.4. Performance of RLC

**Figure 10:**
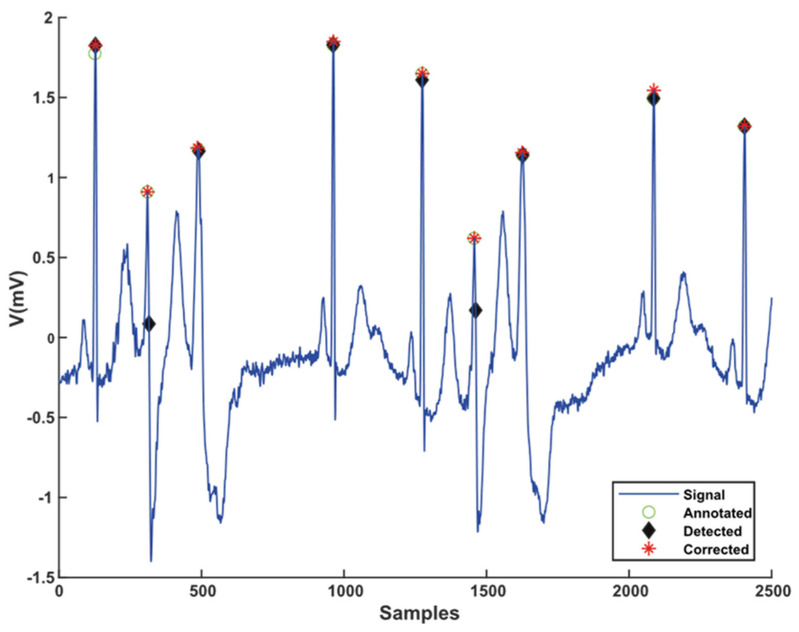
Comparison of detected, corrected, and annotated location of R peaks in sample signal.

The peaks are detected from an integral function of instantaneous energy. During the signal decomposition, the exact locations of peaks are slightly shifted due to the delay of the Wiener filter used in VMD. The RLC algorithm updates the position of the detected R to the exact place. The output of the RLC algorithm is presented in Figure 10.

This study observed that a few R peaks are detected at inaccurate positions. Despite the correction of the detected position, errors still exist. For measuring the RLC algorithm’s performance, this study calculates MMR, SDNN, and RMSSD from R-R intervals extracted from R peaks. A precise “R” position is required to compute R-R distance because it is used to compute the heart rate. The heart rate variability is a vital diagnosis measurement for analyzing CVDs. The mean R-R intervals (MRR), the standard deviation of R-R intervals (SDNN), and the root mean square of R-R intervals are three significant parameters for evaluating the R positional accuracy. 

The proposed approach yielded an error of 0.0048, 0.0276, and 0.042 in MRR, SDNN, and RMSSD, comparing physicians.

### 3.5. Performance Comparison

The proposed method has shown significant performance improvement with an accuracy of 99.94%, which has a sensitivity of 99.98%, positive predictability of 99.96%, and a detection error rate of 0.06%. The results are comparable to the best results published in the literature regarding classification performance and HRV. The proposed method’s success on the MIT-BIH arrhythmia database compared to other existing methods is in Table 5.

## 4. Conclusions

This proposed method’s novelties are fourfold: (1) The proposed DMSE algorithm extracts the best integral function of QRS energy from the ECG signal. (2) The proposed AWS algorithm improved in extracting features of the QRS by suppressing the possibility of mixing the P and T waves features and enhancing the QRSs’ feature only. (3) The proposed RLC algorithm improves R position accuracy. (4) As a result of the DMSE and AWS algorithm, this method significantly improves classifying “R” peaks in the ECG signal. The VMD and HT are used to extract the frequency and energy adaptively and non-recursively. The accuracy of the power spectrum is very high, except for some edge errors. This study overcomes the mode selection by the proposed DMSE algorithm and edge errors by the dropout method. The feature was extracted from a localized position, and the proposed AWS algorithm fixed the window size adaptively. Then, sixteen decision trees are ensembled to distinguish R peaks from other peaks. Finally, a location correction method is used to update to the actual location. The proposed method achieved better performance in sensitivity of 99.98%, the precision of 99.96%, DER of 0.06%, and accuracy of 99.94% than other existing methods.

## Figures and Tables

**Table 1:** Extracted features list.

Domain	Features	Description/Formula	Notation
**Peak** **Features**	Height	The instantaneous energy at a peak location.	$F_{h}^{P}$
	Frequency	The instantaneous frequency at a peak location.	$F_{f}^{P}$
	Prominence	The prominence of a peak	$F_{p}^{P}$
	Width	The width of a peak	$F_{w}^{P}$
**Distance Features**	Previous Peak Distance	$PPD=Peak_{i}^{location}-Peak_{i-1}^{location}$	$F_{p}^{D}$
	Forward Peak Distance	$FPD=Peak_{i+1}^{location}-Peak_{i}^{location}$	$F_{f}^{D}$
**Statistical Features of a Window in Frequency and energy**	Mean	$\mu =\frac{\sum_{i=1}^{N}x_{i}}{N}$	$F_{\mu }^{E}$
			$F_{\mu }^{f}$
	Standard Deviation	$\sigma =\sqrt{\frac{\sum_{i=1}^{N}{\left(x_{i}-\mu \right)}^{2}}{N}}$	$F_{\sigma }^{E}$
			$F_{\sigma }^{f}$
	SNR	$SNR=\frac{\mu }{\sigma }$	$F_{snr}^{E}$
			$F_{snr}^{f}$
	RMS	$r.m.s=\sqrt{\frac{\sum_{i=1}^{N}x_{i}{}^{2}}{N}}$	$F_{rms}^{E}$
			$F_{rms}^{f}$
	Power	$P=\frac{1}{N}\overset{N}{\underset{i=1}{\sum }}x_{i}{}^{2}$	$F_{p}^{E}$
			$F_{p}^{f}$
	Area Under Curve	$A=\int_{i=1}^{N}x_{i}dx$	$F_{a}^{E}$
			$F_{a}^{f}$
	Entropy	$H\left(x\right)=-\overset{N}{\underset{i=1}{\sum }}P(x_{i})logP\left(P(x_{i}\right)$	$F_{h}^{E}$
			$F_{h}^{f}$
	Kurtosis	$K=\frac{\sum_{i=1}^{N}{\left(x_{i}-\mu \right)}^{4}}{\left(N-1\right)\times \sigma^{4}}$	$F_{k}^{E}$
			$F_{k}^{f}$
	Skewness	$S=\frac{\Sigma_{i=1}^{N}{\left(x_{i}-\mu \right)}^{3}}{\left(N-1\right)\times \sigma^{3}}$	$F_{s}^{E}$
			$F_{s}^{f}$
**Signal**	SNR	$SNR=\frac{R^{e}}{R^{n}}$	$F_{snr}^{S}$

**Table 2:** Performance of DMSE algorithm.

Beat Types [32]	MIT-BIH	Experimental	Lost Peaks
False-R	–	22,145	–
Normal	75,052	75,007	45
LBBB	8075	8075	0
RBBB	7259	7259	0
APC	2546	2545	1
Aberrated Atrial Premature	150	149	1
Nodal Premature	83	83	0
Ectopic	2	2	0
Premature Ventricular Contraction	7130	7124	6
Fusion	803	802	1
Atrial Escape	16	16	0
Nodal Escape	229	229	0
Ventricular Escape	106	106	0
Paced	7028	7023	5
Fusion of Paced and Normal	982	982	0
Unclassifiable Beat	33	33	0
Total	109,494	131,580	59

**Table 3:** Confusion matrix for validation, test, and MIT-BIH.

Dataset	TP	FP	TN	FN
10-fold validation set	87,436	141	17,575	112
Test set	21,871	34	4395	16
MIT-BIH	109,415	43	22,102	20

**Table 4:** Performance of classification.

Evaluation Parameters	Validation (%)	Test (%)	MIT-BIH (%)
Sensitivity	99.87	99.93	99.98
Specificity	99.20	99.23	99.81
Precision	99.84	99.84	99.96
Recall	83.26	83.27	83.19
DER	0.29	0.23	0.06
Accuracy	99.71	99.77	99.94
AUC	99.54	99.58	99.89

**Table 5:** Comparison with other methods.

Method	Beats	S (%)	P (%)	D (%)	A (%)	MRR	SDNN	RMSSD
Proposed	131,580	99.98	99.96	0.06	99.940	0.8212	0.1380	0.1715
WTSEE, 2017 [11]	109,494	99.93	99.91	0.163	99.838	0.808	0.145	0.193
ISEE, 2016 [33]	109,532	99.95	99.89	0.159	99.841	-	-	-
STSE, 2014 [34]	108,494	99.84	99.91	0.247	99.753	-	-	-
PSEE, 2013 [27]	109,494	99.92	99.92	0.168	99.832	-	-	-
SEHT, 2012 [35]	109,496	99.93	99.87	0.200	99.800	-	-	-
DOM, 2008 [21]	109,809	99.95	99.85	0.204	99.796	-	-	-
WT, 2004 [13]	109,428	99.80	99.86	0.342	99.288	-	-	-
PT, 1985 [5]	109,809	99.54	99.75	0.717	99.288	0.8174	0.1458	0.1786
Annotated [31]	109,494	-	-	-	-	0.8222	0.1734	0.2206

## Data Availability

The MIT-BIH arrhythmia database can be found at the following website: https://physionet.org/content/mitdb/1.0.0/ (accessed on 8 February 2021).

## References

[1] Heron M. (2016). Deaths: Leading Causes for 2013.

[2] Fuster V., Kelly B.B., Vedanthan R. (2011). Promoting Global Cardiovascular Health. Circulation.

[3] Cook D.A., Oh S.-Y., Pusic M.V. (2020). Accuracy of Physicians’ Electrocardiogram Interpretations. JAMA Intern. Med..

[4] Silva P., Luz E., Silva G., Moreira G., Wanner E., Vidal F., Menotti D. (2020). Towards better heartbeat segmentation with deep learning classification. Sci. Rep..

[5] Pan J., Tompkins W.J. (1985). A Real-Time QRS Detection Algorithm. IEEE Trans. Biomed. Eng..

[6] Chen A., Zhang Y., Zhang M., Liu W., Chang S., Wang H., He J., Huang Q. (2020). A Real Time QRS Detection Algorithm Based on ET and PD Controlled Threshold Strategy. Sensors.

[7] Kumar A., Ranganatham R., Komaragiri R., Kumar M. (2018). Efficient QRS complex detection algorithm based on Fast Fourier Transform. Biomed. Eng. Lett..

[8] Uchaipichat N., Inban S. (2010). Development of QRS Detection using Short-time Fourier Transform based Technique. Int. J. Comput. Appl..

[9] Yue Y., Chen C., Liu P., Xing Y., Zhou X. (2021). Automatic Detection of Short-Term Atrial Fibrillation Segments Based on Frequency Slice Wavelet Transform and Machine Learning Techniques. Sensors.

[10] Gupta V., Mittal M. (2019). QRS Complex Detection Using STFT, Chaos Analysis, and PCA in Standard and Real-Time ECG Databases. J. Inst. Eng. Ser. B.

[11] Park J.-S., Lee S.-W., Park U. (2017). R Peak Detection Method Using Wavelet Transform and Modified Shannon Energy Envelope. J. Healthc. Eng..

[12] Rodriguez Jorge R., García E.M., Córdoba R.T., Bila J., Mizera-Pietraszko J. (2018). Adaptive Threshold, Wavelet and Hilbert Transform for QRS Detection in Electrocardiogram Signals.

[13] Martínez J.P., Olmos S., Laguna P. (2000). Evaluation of a wavelet-based ECG waveform detector on the QT database. Comput. Cardiol..

[14] Ramakrishnan A.G., Saha S. (1997). ECG coding by wavelet-based linear prediction. IEEE Trans. Biomed. Eng..

[15] Lin H.-Y., Liang S.-Y., Ho Y.-L., Lin Y.-H., Ma H.-P. (2014). Discrete-wavelet-transform-based noise removal and feature extraction for ECG signals. IRBM.

[16] Kadambe S., Murray R., Boudreaux-Bartels G. (1999). Wavelet transform-based QRS complex detector. IEEE Trans. Biomed. Eng..

[17] Yochum M., Renaud C., Jacquir S. (2016). Automatic detection of P, QRS and T patterns in 12 leads ECG signal based on CWT. Biomed. Signal Process. Control..

[18] Tan X., Chen X., Hu X., Ren R., Zhou B., Fang Z., Xia S. (2014). EMD-Based Electrocardiogram Delineation for a Wearable Low-Power ECG Monitoring Device. Can. J. Electr. Comput. Eng..

[19] Lu X., Pan M., Yu Y. (2018). QRS Detection Based on Improved Adaptive Threshold. J. Healthc. Eng..

[20] Yazdani S., Vesin J.-M. (2016). Extraction of QRS fiducial points from the ECG using adaptive mathematical morphology. Digit. Signal Process..

[21] Yeh Y.-C., Wang W.-J. (2008). QRS complexes detection for ECG signal: The Difference Operation Method. Comput. Methods Programs Biomed..

[22] Khalaf A.F., Owis M.I., Yassine I.A. (2015). A novel technique for cardiac arrhythmia classification using spectral correlation and support vector machines. Expert Syst. Appl..

[23] Vulaj Z., Brajovic M., Draganic A., Orovic I. Detection of irregular QRS complexes using Hermite transform and support vector machine. Proceedings of the 59th International Symposium ELMAR-2017.

[24] Jun T.J., Park H.J., Minh N.H., Kim D., Kim Y.-H. (2016). Premature Ventricular Contraction Beat Detection with Deep Neural Networks. Proceedings of the 15th IEEE International Conference on Machine Learning and Applications (ICMLA).

[25] Neela T., Namburu S. (2021). ECG signal classification using capsule neural networks. IET Netw..

[26] Yuen B., Dong X., Lu T. (2020). Detecting Noisy ECG QRS Complexes Using WaveletCNN Autoencoder and ConvLSTM. IEEE Access.

[27] Zhu H., Dong J. (2013). An R-peak detection method based on peaks of Shannon energy envelope. Biomed. Signal Process. Control..

[28] Li C., Zheng C., Tai C. (1995). Detection of ECG characteristic points using wavelet transforms. IEEE Trans. Biomed. Eng..

[29] Dragomiretskiy K., Zosso D. (2013). Variational Mode Decomposition. IEEE Trans. Signal Process..

[30] Abeysekera R.M.S.S., Bolton R., Westphal L., Boashash B. Patterns in Hilbert transforms and Wigner-Ville distributions of electrocardiogram data. Proceedings of the ICASSP ‘86. IEEE International Conference on Acoustics, Speech, and Signal Processing.

[31] Moody G., Mark R. (2001). The impact of the MIT-BIH Arrhythmia Database. IEEE Eng. Med. Boil. Mag..

[32] Das M.K., Ari S. (2014). ECG Beats Classification Using Mixture of Features. Int. Sch. Res. Not..

[33] Rakshit M., Panigrahy D., Sahu P.K. (2016). An improved method for R-peak detection by using Shannon energy envelope. Sadhana.

[34] Zidelmal Z., Amirou A., Ould-Abdeslam D., Moukadem A., Dieterlen A. (2014). QRS detection using S-Transform and Shannon energy. Comput. Methods Programs Biomed..

[35] Manikandan M., Soman K. (2011). A novel method for detecting R-peaks in electrocardiogram (ECG) signal. Biomed. Signal Process. Control..

